# Allergy-associated T cell epitope repertoires are surprisingly diverse and include non-IgE reactive antigens

**DOI:** 10.1186/1939-4551-7-26

**Published:** 2014-10-22

**Authors:** April Frazier, Veronique Schulten, Denise Hinz, Carla Oseroff, John Sidney, Bjoern Peters, Alessandro Sette

**Affiliations:** La Jolla Institute for Allergy & Immunology, 9420 Athena Circle, La Jolla, CA 92037 USA

**Keywords:** T cells, Specific immunotherapy, Timothy grass, Cytokine, Epitopes

## Abstract

We recently identified T cell epitopes associated with human allergic responses. In a majority of cases, responses focused on a few immunodominant epitopes which can be predicted on the basis of MHC binding characteristics. Several observations from our studies challenged the assumption that T cell epitopes are derived from the same allergen proteins that bind IgE. Transcriptomic and proteomics analysis identified pollen proteins, not bound by IgE. These novel Timothy Grass proteins elicited vigorous Th2 responses, suggesting that unlinked T cell help is operational in pollen-specific responses. Thus, the repertoire of antigens recognized by T cells is much broader than IgE-binding allergens. Additionally, we evaluated the use of epitopes from these novel antigens to assess immunological changes associated with Specific Immunotherapy (SIT). We found that a marked decrease in IL5 production is associated with clinically efficacious SIT, suggesting that these novel antigens are potential immunomarkers for SIT efficacy.

**Electronic supplementary material:**

The online version of this article (doi:10.1186/1939-4551-7-26) contains supplementary material, which is available to authorized users.

## Introduction

Herein we will briefly summarize data generated in our laboratory pertaining to the identification of T cell epitopes associated with allergic responses in humans. We will further discuss this data in the context of the potential use of allergen-derived T cell epitopes as biomarkers of specific immunotherapy (SIT).

These studies were prompted by the observation that T cells contribute to pathology associated with allergy both directly and indirectly through modulation of immune responses [1]. The involvement of T cells in SIT has been reviewed recently [2–4], and it has been suggested that epitopes may provide a tool to monitor disease and therapeutic intervention. Furthermore, several studies have forwarded the hypothesis that T cell epitopes themselves have potential as immunotherapeutic vaccine components [5, 6]. Yet, for the majority of the most common allergens, the corresponding T cell epitopes have not been thoroughly mapped.

Our approach and results will be articulated in three main steps. First, we will review the development of techniques and general approaches for the identification of allergen-derived epitopes and their application to some of the most common allergen sources. Second, we will describe a series of studies that questioned the assumption that T cell epitopes are derived from the same allergen proteins that bind IgE. The final series of experiments described here will relate to the use of epitopes to assess immunological changes associated with SIT maintenance phase.

### A general strategy for epitope identification and characterization

A number of different methods are available for the identification and characterization of peptide epitopes recognized by human class II restricted T cells. They include generation of stable T cell lines and clones, isolation of naturally processed ligands, screening of libraries of overlapping peptides, and generation of mimotope libraries and prediction of HLA class II binding capacity by bioinformatics analysis.

Our group in particular has been interested in the use of bioinformatics predictions combined with experimental tests of predicted binders using lymphocytes derived from allergic patients [7–10]. One issue that has to be considered in the implementation of this approach is the fact that HLA class II molecules are very heterogeneous. They are in fact encoded by four different loci (HLA polygeny), and at each of these loci hundreds of allelic variants are represented in the general population (HLA polymorphism) [11–14].

To address this challenge, we put forth the hypothesis that while the large number of HLA class II variants would likely be reflected in a large degree of heterogeneity of responses, a large fraction of the response might be accounted for by a limited number of more dominant epitopes. We further hypothesized that T cell epitopes capable of cross-binding multiple HLA class II molecules (i.e., promiscuous epitopes) would likely be amongst such dominant epitopes. Hence, we suggest that predicting promiscuous HLA class II epitopes might offer a practical means to identify a rather significant fraction of T cell allergen epitope specificities.

### Validation of the approach

The potential merit of the hypothesis formulated above was tested experimentally in the Timothy Grass (TG) system [10]. For these studies, we considered all TG proteins considered allergens by IUIS (Phl p 1, 2, 3, 4, 5, 6, 7, 11, 12, 13). It is important to note that these allergens were previously defined based on their capacity to elicit IgE reactivity. To ensure we would comprehensively detect T cell responses directed against these allergens, a series of 687 15-mer peptides overlapping by 10 residues were synthesized to completely span each protein sequence. Peptide pools were tested by ELISPOT for reactivity using short-term T cell lines obtained by *in vitro* restimulation with TG extract. Positive pools were subsequently deconvoluted to identify the individual 15-mer(s) eliciting allergen-specific responses.

Upon completion of the screen that included 10 non-allergic donors and 33 allergic donors, 8 of which were in maintenance phase of SIT, we noted, as expected, a large degree of heterogeneity of responses. At least 43 different epitopes/antigenic regions were defined. However, we also noted that a few regions accounted for a disproportionately large fraction of the responses. In fact, just the top nine regions accounted for over half of the total response. Subsequent analysis verified that these epitopes were indeed promiscuous (i.e., they were capable of eliciting responses in the context of multiple HLA). Further analysis also revealed that binding predictions could identify the most promiscuous epitopes accounting for a relatively large fraction of the responses.

Based on these results, we developed a predictive strategy to identify specifically those peptides binding to multiple HLA. We applied this strategy to a larger scale effort targeting epitope identification for an additional set of 28 allergen sources [7, 9, 15]. These included cockroach antigens and various tree, grass, fungi, and animal allergens. Our selection of these sources was based on a focus towards allergens that were relatively common at the two clinical sites participating in the study and for which allergen sequences were available from IUIS. The predictive strategy validated in the TG model system was utilized and a total of over 250 different antigenic regions were identified. To the best of our knowledge, this study reported the first-ever human T cell epitopes from 16 of these common allergen sources.

In conclusion, our studies have found that in a majority of cases allergen-specific responses are directed against relatively few immunodominant epitopes. Further, these epitopes can be identified and/or predicted on the basis of MHC binding characteristics.

### Are T cell epitopes derived from the same allergen proteins that bind IgE?

It was generally assumed that T cell epitopes are derived from the same allergens that bind and elicit IgE responses. However, in the course of the studies described above, we made two observations that led us to question the validity of this assumption.

First, in both the TG and cockroach allergy systems, we observed that T cell responses and IgE responses did not correlate at the level of individual patients [9, 10]. More specifically, while in general the magnitude of T cell responses and IgE titers directed against allergen extracts were positively correlated, a different picture was noted in the case of individual recombinant allergens. That is, in several patients, a vigorous IgE response might be detected against antigens for which no T cell response was detected while, conversely, in the same patient the dominant T cell response was detected against a different antigen not dominantly targeted by IgE.

Second, in several patients, no T cell response could be detected against any of the known IgE-binding allergens, despite vigorous T cell responses to the TG extract. We hypothesized that these observations might be explained by T cell responses targeting additional pollen proteins other than the known allergens. The absence of an exact correlation of T and B cell responses on the antigen level is not really surprising. In fact, this notion, called “antigen bridge” [16–21], while not experimentally explored in the case of allergen specific responses, is “textbook immunology” when, for example, infectious diseases are considered. In this case, it is well appreciated that helper T cells specific for internal proteins of common viruses (influenza, hepatitis B) provide effective help to B cells specific for antigens expressed on the virus surface.

### Transcriptomic and proteomic analysis of TG to test the breadth of the TG epitope repertoire

**Figure 1:**
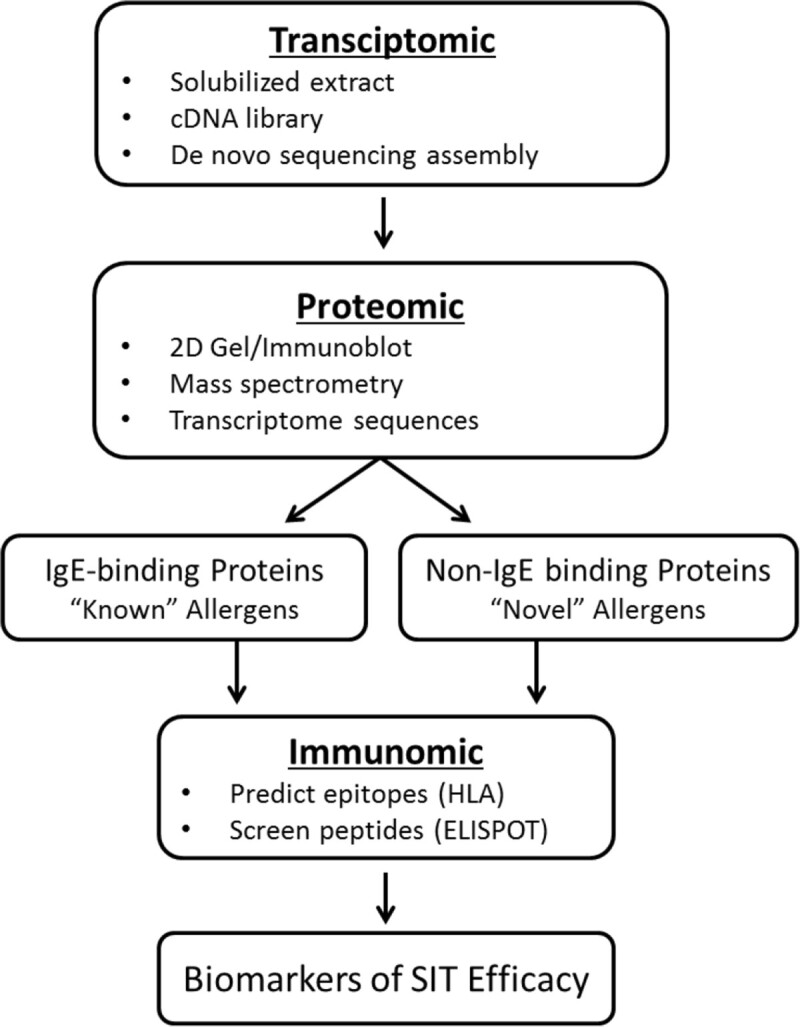
**Scheme of epitope identification process.** Diagrammatic representation of the process of antigen, epitope, and biomarker identification as detailed in this review.

Based on these data, we hypothesized that Th2 responses in allergic patients might recognize antigens contained in the pollen particles but not necessarily limited to the ones bound by IgE. Testing this hypothesis required a multidisciplinary approach (Figure 1), including a transcriptomic analysis based on obtaining mRNA sequences from TG pollen. This analysis was coupled with a proteomics analysis, which identified pollen proteins by mass spectrometry (MS) from extract using the transcriptome as a reference. Together, these two analyses enabled the immunomics analysis in which peptides binding promiscuously to HLA class II molecules were predicted, synthesized and tested for T cell recognition.

Identification of novel proteins was accomplished by isolating TG pollen mRNA and analyzing high-throughput sequencing from an Illumina Genome Analyzer. A total of 1,016,285 unique, putative transcripts (including allelic variants and isoforms) were identified. Next, we used gel separation and MS analysis to identify which of the newly identified transcripts encoded proteins detectable in TG pollen. More specifically, pollen extract was separated on 2D gels and specific spots were picked for further analysis based on antibody or protein staining. These spots were then cut out from the gel and analyzed by mass spectrometry. As a result, 83 new proteins derived from the 2D gel and an additional ten proteins derived from whole extract MS analysis were chosen for further studies [8].

To identify T cell epitopes on the basis of HLA class II binding predictions, we predicted the capacity of peptides derived from the novel proteins to bind a panel of 25 common HLA class II DR, DP, and DQ molecules selected to represent the most prevalent alleles in the general world-wide population, inclusive of all major ethnicities [12]. A set of 822 peptides predicted promiscuous binders (i.e., >12 HLA variants predicted to be bound) was then synthesized and tested for IL5 production in PBMC from TG allergic donors.

When compared to known allergens, the results clearly indicated that a majority of Th2 responses in TG allergic individuals target epitopes derived from novel antigens, some of which are not targeted by IgE responses. Further experiments confirmed this discovery by showing that memory T cells are the source of IL5 T cell responses after *in vitro* expansion, and showing that responses to both conventional and novel TG antigens can be detected directly *ex vivo*.

In conclusion, the results summarized in this section indicate that novel TG proteins elicited Th2 responses, explaining the reactivity gap between known allergens and whole extract. IL5 production was directed to several antigens not targeted by IgE or IgG, suggesting that unlinked T-cell help is operational in pollen-specific responses. In the end, the universe of T cell inducing antigens was found to be much broader than that delineated by IgE-binding allergens.

### Immunological changes associated with SIT maintenance phase

Despite unquestionable evidence demonstrating that SIT is effective in treating allergic disease [22–25], several limitations are also associated with this treatment modality. Safety considerations dictate a rather laborious regimen of administration. The same considerations limit the dosing, potentially also limiting the effectiveness of the treatment. Various alternative treatment modalities are being considered, but their design and testing has been hampered by an incomplete understanding of mechanisms of action and by the lack of immunomarkers of efficacy [26, 27]. These limitations in turn have slowed the process of design and testing of alternatives, potentially inhibiting the development of improved immunotherapies.

With these considerations in mind, we initiated a comparison of cytokine production in allergic and SIT donors [28]. In these comparisons, we utilized both known and novel TG antigens. Epitope specific responses were analyzed in a cross-sectional study utilizing ELISPOT assays specific for IL5, IL10, IFN-gamma and IL17. The results of these experiments revealed, for both known and novel allergens/antigens, a marked decrease in IL5 and no difference in IL10 production for the SIT donors. The decrease in IL5 was expected, as this was chosen as representative of Th2 cytokines. Conversely, the lack of effect in terms of IL10 was somewhat unexpected in light of previous observations [29, 30], and might be related to the fact that IL10 production has been reported to be an early and transient event associated with early SIT phases (unlike the maintenance phase associated with the SIT donors analyzed here).

When epitopes from known and novel antigens were compared, the decrease in IL5 production associated with SIT was similar, and, if anything, more pronounced in the case of the novel antigens and epitopes. Taken together, these data have suggested several potential immunomarkers for SIT efficacy as well as possible approaches towards defining a mechanism of action.

### A subset of NTGAs for which IL5 responses are most down-regulated in SIT

Based on these results, we selected a subset of novel Timothy Grass antigens (NTGAs) and epitopes for which the IL-5 response was found to be particularly down-modulated in SIT and used it for additional studies in new donors [28]. An important consideration with respect to the relevance of these antigens and epitopes for their potential use in therapeutic applications is that several of them are not targeted by IgE, and are thus expected to offer a safer alternative for immunotherapy. When these epitopes were tested in a new donor cohort of SIT and allergic controls, the drastic decrease in IL5 production was replicated. Concomitantly, a highly significant increase in IFN-gamma production was noted for these selected epitopes when SIT donors were compared to untreated allergic patient controls. The lack of change in the levels of IL10 production noted in the previous cohort was also reproduced. Finally, additional experiments demonstrated that the reduction of Th2 responses in SIT donors is not limited to IL5, but also extends to other Th2 cytokines such as IL4 and IL13.

### Modulation of cytokine responses to the MNA pool correlates with SIT efficacy

In the course of recruitment of the SIT donors utilized in these experiments, each donor provided a subjective evaluation as to whether or not the treatment had helped to alleviate their symptoms. When the data pertaining to the modulation of the IL5 response was subsequently analyzed, it was found that modulation of IL5 responses were much more pronounced in the donors self-reporting benefit from treatment [28]. That is, IL5 responses in the group of individuals that did not benefit from SIT were essentially indistinguishable from those of allergic untreated donors, while the responses seen in the group of SIT donors that subjectively reported to have benefitted from treatment were significantly lower.

These data raise the possibility that T cell responses to certain modulated novel antigen (MNA) epitopes can be used as biomarkers of SIT efficacy. However, additional studies are clearly required, including those addressing larger patient cohorts as well as longitudinal studies (e.g., samples from the same patient during the course of SIT treatment) and those incorporating more objective evaluations of SIT clinical efficacy. These subsequent studies should also evaluate whether modulation of Th2 responses to specific epitopes follows or precedes the clinical benefit, and determine how long the modulation lasts. These studies should also correlate changes in epitope specific responses with other immunological changes that have been proposed as biomarkers of SIT efficacy, such as increases in IgG4 titers.

### Synopsis

We utilized a bioinformatics-based approach to characterize T cell responses to common allergens. Peptides from novel, non-IgE-binding proteins elicited Th2 responses, suggesting that unlinked T-cell help is operational in pollen-specific responses. Thus, the repertoire of T cell allergens is much broader than that defined by IgE-binding. IL5 responses to these novel antigens from SIT donors that subjectively reported to have benefitted from treatment were significantly lower than those of individuals that did not benefit from SIT. These observations raise the possibility that allergen specific epitopes can be used as biomarkers of SIT efficacy.

## Authors’ original submitted files for images

Below are the links to the authors’ original submitted files for images. Authors’ original file for figure 1

## Abbreviations

IL: Interleukin
SIT: Specific immunotherapy.
