# Supplementary figures and images for: Allergy-associated T cell epitope repertoires are surprisingly diverse and include non-IgE reactive antigens
Source: World Allergy Organ J. 2014 Oct 22;7(1):26. doi: 10.1186/1939-4551-7-26 (PMC4210551; doi:10.1186/1939-4551-7-26)

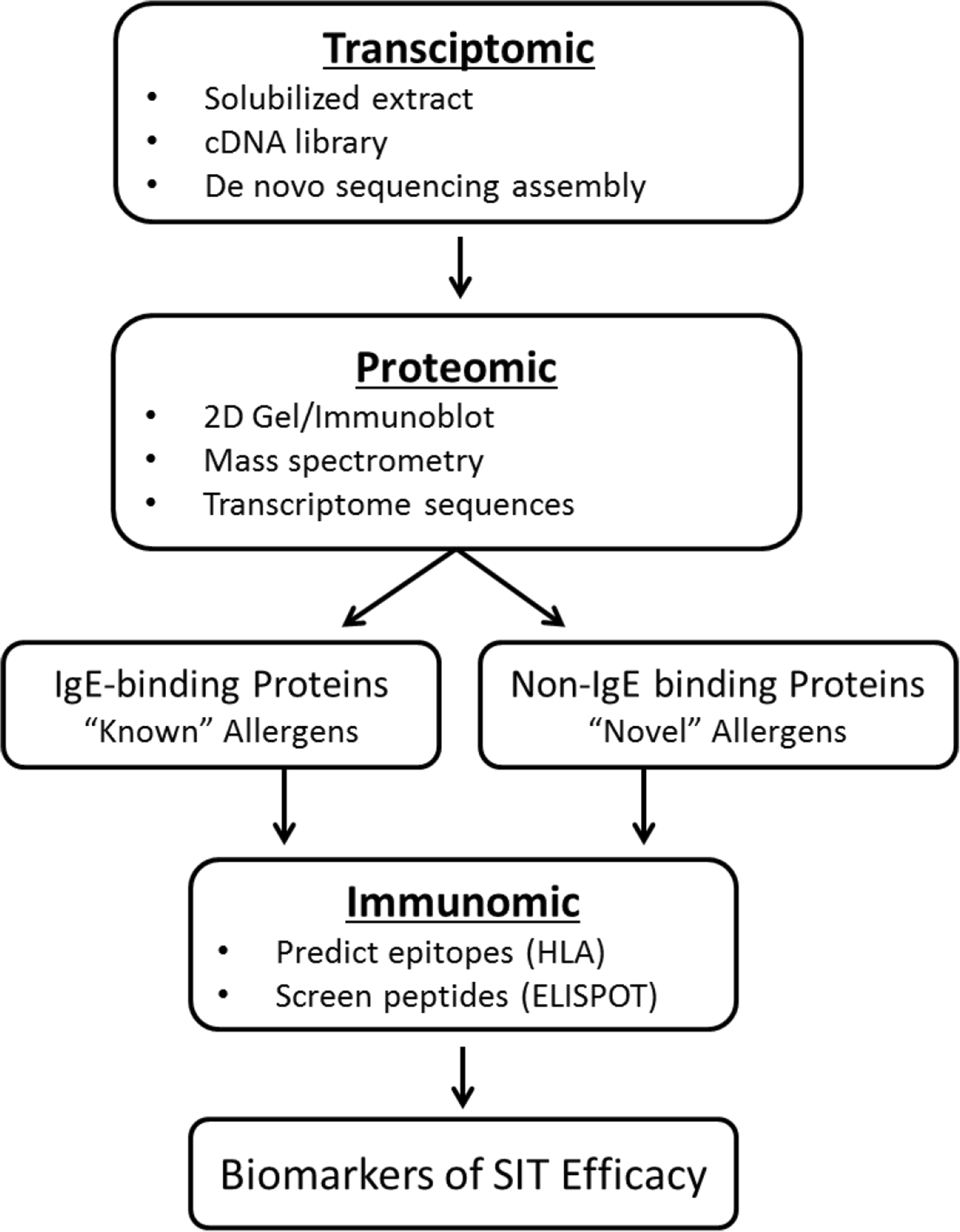

Supplement: Supplementary file 1 — Authors’ original file for figure 1 [file 40413_2014_68_MOESM1_ESM.tiff]
